# Analysis of Vertical Micro Acceleration While Standing Reveals Age-Related Changes

**DOI:** 10.3390/geriatrics5040105

**Published:** 2020-12-18

**Authors:** Tadayoshi Minamisawa, Noboru Chiba, Kaori Inoue, Tatsuya Nakanowatari, Eizaburo Suzuki

**Affiliations:** 1 Department of Physical Therapy, Yamagata Prefectural University of Health Sciences, 260 Kamiyanagi, Yamagata 990-2212, Japan; tnakanowatari@yachts.ac.jp (T.N.); esuzuki@yachts.ac.jp (E.S.); 2 Department of Occupational Therapy, Yamagata Prefectural University of Health Sciences, 260 Kamiyanagi, Yamagata 990-2212, Japan; nchiba@yachts.ac.jp (N.C.); kainoue@yachts.ac.jp (K.I.)

**Keywords:** quiet standing, vertical center of mass acceleration, continuous wavelet transform, power spectral density, aging

## Abstract

In this study, we investigated the fluctuation characteristics of micro vertical acceleration of center of mass (vCOMacc) in standing and examined the usefulness of vCOMacc as an aging marker for standing control abilities. Sixteen young and 18 older adults participated in this experiment. Data for vCOMacc were calculated as the vertical ground reaction force value divided by each participant’s body mass using a force plate. The COMacc frequency structure was determined using the continuous wavelet transform to analyze the relative frequency characteristics. For time domain analysis, we determined the root mean square (RMS) and maximum amplitude (MA) of the integrated power spectral density. We also analyzed the correlation between vCOMacc and lower limb muscle activity. The relative frequency band of vCOMacc was higher in older than young adults, and the time domain indicators were sufficient to distinguish the effects of aging. Regarding the relationship between vCOMacc during standing and muscle activity, a correlation was found with the soleus muscle in young adults, while it was moderately correlated with the gastrocnemius muscle in older adults. The cause of vCOM may be related to differences in muscle activity, and vCOMacc may be utilized to more easily assess the effects of aging in standing control.

## 1. Introduction

Stable posture control is essential for safety in daily life and may determine quality of life, especially for older adults. To evaluate bipedal standing ability, many studies use force plates to analyze center of pressure (COP) [1,2,3,4,5] or center of mass (COM) acceleration [6,7,8,9]. The COP trajectory has been shown to be able to predict the dynamic response of attitude control systems to perturbations of young or older adults even during a quiet stance [10,11]. Alternatively, anterior-posterior center of mass acceleration (apCOMacc) [6,7,8,9] can be used to analyze the aging, developmental, and disease characteristics in a controlled standing system. In this regard, such devices are useful devices that can be easily measured, leading to many studies, especially on sagittal plane trajectory [12,13,14]. On the other hand, vertical fluctuation is much smaller than horizontal fluctuation; thus, there is generally little interest in such variation characteristics [15]. However, it has been reported that the vertical ground reaction force (Fz) parameter allows the collection of data at a lower cost, because it only collects the weight load fluctuation of the individual; additionally, it is more robust against fluctuations between trials [16]. If an analysis method using only vertical acceleration can be proposed for balance control due to aging, clinical evaluation using a simpler and lower-cost device will be possible. In a previous study, the standard deviation of Fz was found to be significantly correlated with clinical balance scale scores, and it was highly reliable for identifying stance tasks compared to COP measurements [17]; however, few studies utilized such force plates to measure the body acceleration (Fz/m, m = body mass) in the vertical direction for the analysis of standing control [18]. Since the Fz component is not well known, its relationship with the key muscles of lower limbs in standing control is also unclear. It would be preferable if such an index could lead to the evaluation of physical function and the identification of abnormalities different from traditional indices [19]. Easy-to-collect biometric information is an advantage for future clinical studies and may provide another aspect for standing control studies. The first purpose of this study was to evaluate whether this simple and micro vibration component can be a balance marker in relation to age-related changes or to the major muscles of postural maintenance. Another concern relates to the method of analysis for biological signals. Fourier transform frequency analysis, used in previous studies, is a common method for analyzing postural sway. Past researchers used fast Fourier transform algorithms to compare postural sway in young adults and older adults, with respect to the power spectral content of COP trajectory [20,21,22]. These analyses assume that the signal being analyzed is stationary. However, this is not the case for most biological signals, which exhibit varying degrees of nonstationarity [23]. Furthermore, it is well known that the gastrocnemius muscle, which is the main muscle for standing control, provides impulsive control during postural sway [24]; hence, its temporal characteristics should also be noted in order to more closely investigate the characteristics of postural stability control [25]. Time–frequency analyses are particularly suitable for analyzing local dynamics at intermittent times, which occur in nonlinear systems [26]. Thus, continuous wavelet transform (CWT) calculations enable a complete study of the attitude control spectrum and time characteristics. Furthermore, wave transformation can provide useful results for identifying changes in attitude control. Previous studies measured COPs using wavelet analysis [26,27]; however, analysis with acceleration as an index is considered a simple and practical parameter [7,8]. The time–frequency analysis of vertical center of mass acceleration (vCOMacc) provides an easy and alternative method for the detection of instantaneous acceleration changes in body sway. It has not yet been debated how such simple signals change with age and what neurological background causes them. Furthermore, it is unknown if vCOMacc is associated with apCOMacc. Therefore, the purpose of this study was to compare the quiet standing behavior of young adults and older adults by examining the time and frequency domain characteristics of vCOMacc.

## 2. Materials and Methods

### 2.1. Subjects

This study included 16 healthy young adults (age: 20.4 ± 0.6 years; height: 165.9 ± 8.3 cm; weight: 58.3 ± 6.3 kg) and 18 healthy older adults (age: 72.9 ± 3.6 years; height: 162.0 ± 8.2 cm; weight: 62.9 ± 8.6 kg), a total of 34 subjects. Exclusion criteria included a history of musculoskeletal injuries or diseases and neurological disorders. All subjects gave their informed oral and written consent for inclusion before they participated in the study. The study was conducted in accordance with the Declaration of Helsinki, and the protocol was approved by the ethics review board of Yamagata Prefectural University of Health Sciences (approval ID: 1310-16).

### 2.2. Measurements

Participants were requested to stand, with their eyes open, on a single force plate (60 × 90 cm, Type 9287A; Kistler, Winterthur, Switzerland). They were instructed to watch a target, which was placed at eye level, while keeping their arms and stance in a comfortable position. Anteriorposterior positioning of the feet was based upon a predetermined distance and was aligned using markers on the force plates. The force plate was used to extract both Fz data and anterior-posterior ground reaction force data (Fx) points. For the purpose of clarifying the relationship between vCOMacc and the muscle activity of the lower limbs, a surface electromyography (EMG) system (TrignoTM Wireless EMG, Delsys Inc., Boston, MA, USA) was used to collect muscle activity data. Prior to attaching the electrodes, skin impedance was reduced by wiping the skin with alcohol swabs. The EMG electrodes were placed on the soleus (So) and medial gastrocnemius muscles (MG) of both limbs. The EMG signals of lower limb muscle were in the range of 10–450 Hz when bandpass-filtered offline (fourth-order Butterworth filter). Each subject underwent three trials, with a sufficient resting period between each trial. All data were collected at a sampling frequency of 1000 Hz, and signals were loaded onto a personal computer for analysis. The duration of each trial was approximately 60 s. Only data from the last 55 s were subjected to subsequent analyses.

### 2.3. Data Analysis

For both Fz and Fx data, analog offset (DC offsets; mean amplitude displacement from zero) were removed. vCOMacc and apCOMacc were obtained by dividing the ground reaction force (GRF) in the Fz and Fx directions by the participant’s body mass. The vCOMacc data were subjected to filtering using a fourth-order Butterworth bandpass filter, which employed a zero-phase lag with a cutoff frequency of 0.1–20 Hz. The apCOMacc data were low-pass-filtered at a frequency of 10 Hz using a Butterworth filter [27].

We used the CWT method to analyze the effect of aging on the time domain and the frequency domain of COMacc. Continuous wavelet transform describes a mathematical technique that can be used to analyze a complex time-series signal with variable power or magnitude over a wide range of frequencies. The CWT, shown in Equation (1), was calculated using AutoSignal software (Systat Software Inc., San Jose, CA, USA). The CWT of a discrete sequence *x_n_* is defined as the convolution of *x_n_* with a scaled and translated version of *ψ*_0_*(n)*.

(1) $$W_{n}\left(s\right)=\overset{N-1}{\underset{n^{\prime }=0}{\sum }}x_{n^{\prime }}\sqrt{\frac{\delta t}{s}}\psi_{0}^{*}\left[\frac{\left(n^{\prime }-n\right)\delta t}{s}\right]$$

where the complex conjugate is indicated by *, *N* is the data series length, and *δt* is the sampling interval. By varying the wavelet scale *s* and translating along the localized time index *n*, it is possible to construct an image showing the amplitude of any feature versus the scale and how this amplitude varies with time. The subscript zero on *ψ* (Equation (2)) was added to indicate that this value of *ψ* was also normalized.

(2) $$\Psi_{0}\left(\tau \right)=\pi^{-\frac{1}{4}}e^{i\sigma \tau }e^{-\frac{\tau^{2}}{2}}$$

where τ is the adjustable, nondimensional time parameter. For this study, the adjustable parameter σ was set to 8 in the AutoSignal environment. The global wavelet spectrum (Equation (3)), which is defined as the time average over a series of wavelet powers, can be expressed as

(3) $${\bar{W}}^{2}\left(S\right)=\frac{1}{N}\overset{N-1}{\underset{n=0}{\sum }}{\left|w_{n}\left(S\right)\right|}^{2}$$

where *W_n_* is the CWT coefficient, and *n* and *N* are the specific time ranges to average the CWT coefficients. A continuous wavelet (Morlet) time–frequency analysis, which determines the integrated power (time-integral squared amplitude (TISA)) across time [28,29,30], was performed. The TISA power is defined as

TISA (Power) = Δt × (*Re*^2^ + *Im*^2^)/2 (4)

where *Re* and *Im* are the real and imaginary parts of the transformed data, respectively, and Δt is the sampling interval (Equation (4)).

#### 2.3.1. Frequency Domain Analysis

The normalized power spectral density (power in frequency bin divided by the total amount of power) was averaged across a 2 Hz band recording, which ranged from 0 to 20 Hz for vCOMacc or 0 to 10 Hz for apCOMacc.

#### 2.3.2. Time Domain Analysis

**Figure 1:**
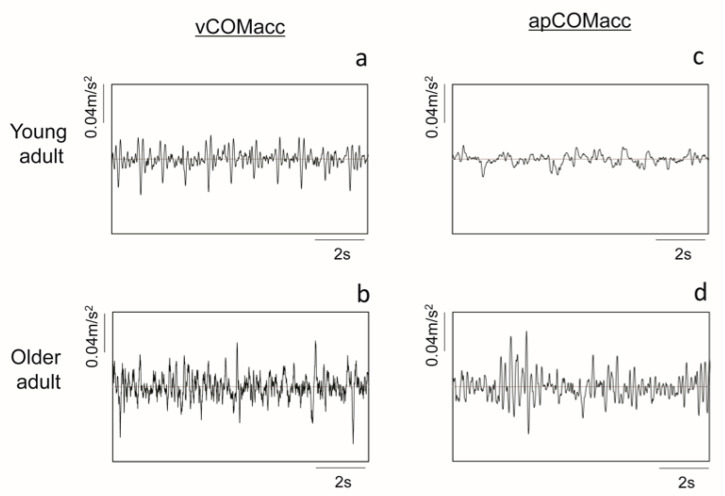
Representative time series of vertical center of mass acceleration (vCOMacc) (left panel—(**a**,**b**)) and anterior-posterior center of mass acceleration (apCOMacc) (right panel—(**c**,**d**)) from a young adult (upper panel) and an older adult (lower panel) for standing.

For each participant’s COMacc and EMG data, the root mean square (RMS) was used to quantify the mean power of COMacc (Figure 1). Measurements of the average power spectral density of COMacc and EMG data were calculated for each trial from the RMS as follows:

(5) $$\mathrm{RMS}=\sqrt{\frac{1}{N}\overset{N}{\underset{i=1}{\sum }}{\left[x\left(i\right)\right]}^{2}}$$

where *x*(*i*) is the COMacc data for sample number *i*, and *N* is the number of samples. Moreover, the maximum amplitude (MA) of the COMacc integrated power was compared for both groups, which allowed for an evaluation of the instantaneous changes in COMacc and an examination of the effects of aging.

### 2.4. Statistical Analysis of COMacc Data

Statistical analysis of the mean power value within each frequency band for the two groups was performed using two-factor analysis of variance, with a statistical significance level set at *p* < 0.05. Time domain vCOMacc and apCOMacc data were statistically tested for both groups (young vs. older adults) using a non-paired t-test. The correlation between RMS vCOMacc or RMS apCOMacc and the correlation between each COM and EMG were estimated using Pearson’s correlation coefficient (*r*) (* *p* < 0.05, ** *p* < 0.01).

## 3. Results

**Figure 2:**
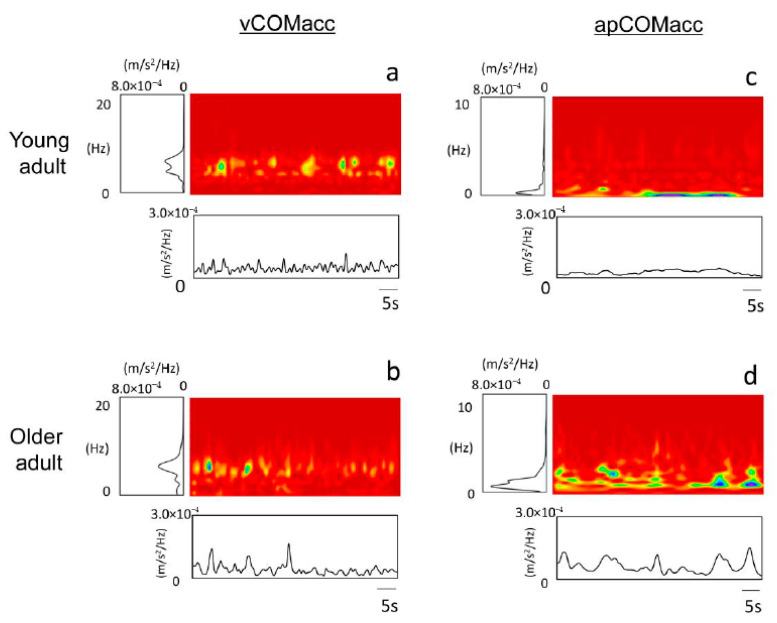
Examples of analysis in two-dimensional plots from continuous wavelet transform (CWT) of COMacc in young adults (panels (**a**,**c**)) and older adults (panels (**b**,**d**)). The power spectral density of the CWT shows the time-integrated square amplitude. Frequency domain analysis was normalized, and the power spectral densities were averaged across the 1 Hz band over the frequency range of 0–20 Hz for vCOMacc and 0–10 Hz for apCOMacc.

Figure 1 shows a representative time-series example of vCOMacc or apCOMacc from young adults (upper panel, a and c) and older adults (lower panel, b and d). Figure 2 shows typical examples of frequency domain and time domain analysis results for each COMacc using CWT. Approximately 90% of vCOMacc data in both young and older adults were present below 10 Hz; thus, those below 10 Hz were analyzed in this study. 

### 3.1. Frequency Domain

**Figure 3:**
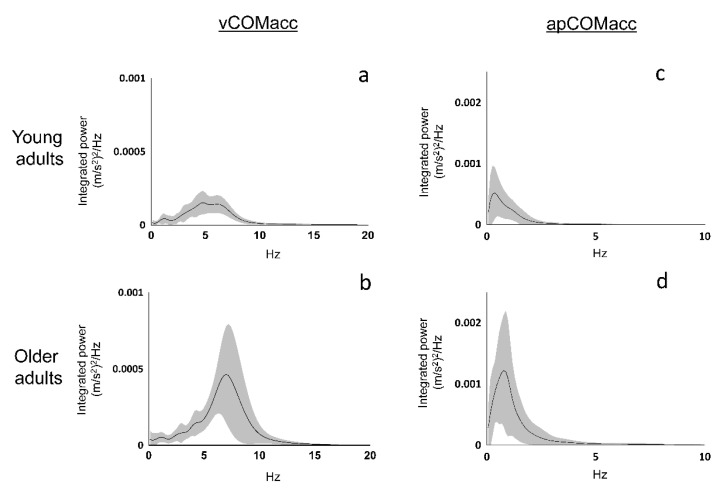
Power spectral density functions of vCOMacc (left panel) and apCOMacc (right panel). The upper and lower panels indicate the results for young adults (panel (**a**,**c**)) and older adults (panel (**b**,**d**)), respectively. The solid line represents the average in each frequency band of the group. The shaded area represents one standard deviation.

Analysis of the frequency structure of COMacc indicated that the power spectrum of vCOMacc was stronger within older adults, mainly with respect to the relative power observed across the 2–8 Hz band. In addition, almost every young adult displayed major peaks in the 4–6 Hz power spectrum, whereas older adults typically showed peaks in the vCOMacc spectrum between frequencies of 6 and 8 Hz (Figure 3, Table 1). Moreover, the main frequency band of apCOMacc in both groups was less than approximately 2 Hz, which was similar to previous reports (Figure 3, Table 2) [7].

### 3.2. Time Domain

**Figure 4:**
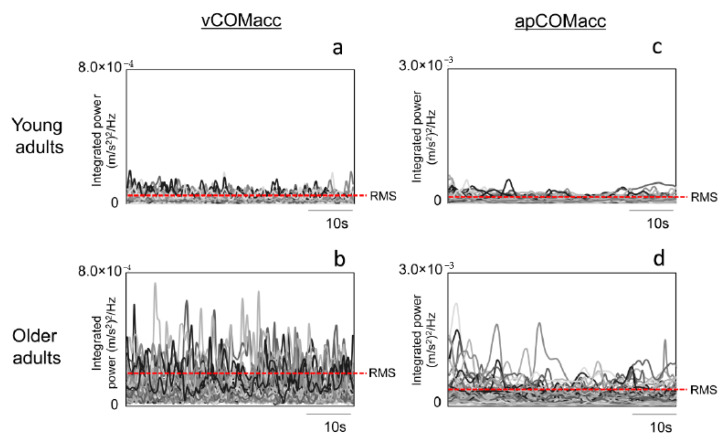
Superposition time-series waveforms of young (panels (**a**,**c**)) and older adults (panels (**b**,**d**)) for vCOMacc (left panel) and apCOMacc (right panel) of the continuous wavelet time–frequency spectrum (time-integral squared amplitude power) during quiet standing. The dotted line in the figure shows the root mean square value.

Wavelet analysis of body acceleration revealed several sudden peaks in the integrated power of vCOMacc. The MA of the integrated power was larger in older adults (young adults: 1.0 × 10^−4^ ± 2.0 × 10^−9^, older adults: 3.0 × 10^−4^ ± 2.0 × 10^−8^; *p* < 0.01) (Table 1). For apCOMacc, young adults displayed an average MA value of 2.0 × 10^−4^ ± 8.0 × 10^−5^, while older adults had an average MA value of 5.0 × 10^−4^ ± 3.0 × 10^−8^ (*p* < 0.01) (Table 2). As presented in Table 1, the group mean value of RMS vCOMacc was significantly greater in older adults than in young adults (young adults: 4.2 × 10^−5^ ± 1.4 × 10^−5^, older adults: 1.0 × 10^−4^ ± 5.8 × 10^−5^; *p* < 0.05) (Figure 4). For apCOMacc, young adults exhibited an average RMS value of 7.9 × 10^−5^ ± 2.8 × 10^−5^, whereas older adults had an average RMS value of 2.0 × 10^−4^ ± 1.0 × 10^−4^ (*p* < 0.01) (Figure 4, Table 2). 

### 3.3. Correlation Analysis for vCOMacc and apCOMacc

There was no significant correlation between vCOMacc and apCOMacc in both young and older adults (young: *r* = 0.16, *p* = 0.56; older adults: *r* = 0.42, *p* = 0.08) (Table 1).

Table 3 and Table 4 show the correlation between vCOMacc or apCOMacc and EMG for young adults and older adults, respectively. In young adults, a high correlation coefficient was obtained with COMacc for the frequency band above 4 Hz. In older adults, a negative correlation was found between vCOMacc and MG in the 0–2 Hz frequency band, while a positive correlation was found between apCOMacc and EMG in the 4–6 and 6–8 Hz frequency bands

## 4. Discussion

The purpose of this study was to apply wavelet spectral analysis to the study of postural control and to determine whether aging results in measurable changes in spectral properties during quiet standing.

### 4.1. Frequency Domain

vCOMacc revealed differing power spectral densities according to age, and the frequencies showed measurable differences between age groups. Specifically, the relative power spectral density was characterized by a high-frequency band in older adults. Physical tremors of the soleus muscle [31] or increased power of lower limb muscle activity in the high-frequency band [32] may be associated with vCOMacc in older adults; therefore, vCOMacc can be an indicator of age-related neurological or musculoskeletal decline. Worthy of note was the correlation between the gastrocnemius muscle and vCOMacc in the lower-frequency band. Previous studies reported that the lower-band oscillatory drive of the bilateral plantar flexors during a quiet stance is characteristic in older adults [33]. Such an increase in the low-frequency bands may be associated with poor motor unit firing rate [34], reduced vibrotactile sensitivity of the foot soles [35], and amplified vestibular motor response [36]. During quiet standing, the activation of calf muscles plays an important role in the postural control of standing balance [37,38], because the ankle joint torque controls the COM behavior [39]. Thus, if the behavior of MG activity can be estimated from a specific frequency band of vCOMacc, it may be possible to easily obtain more information from a simple bio signal.

### 4.2. Time Domain

The results obtained from the MA RMS and the integrated power spectral density RMS were enough to reliably distinguish young adults from older adults, as shown in Figure 2. A significant increase in the power spectral density of vCOMacc during standing may be associated with the age-related decrease in dynamic ballistic muscle strength [40] or in the number of motor units [41]. Additionally, a persistent increase in spectrum density in older adults may be associated with the sustained and enhanced muscle activity of the plantaris flexors [42,43], as well as a decline in cutaneous feedback and proprioception [44]. Otherwise, during standing, the body is constantly subjected to micro vibrations, which may be caused by myocardial contractions [45] or physiological tremors [46,47,48].

Previous studies also investigated the amplitude and structural variabilities of apCOMacc and EMG, showing an enhanced relationship between postural variability and responsible muscle activation [49] Similarly, this study also evaluated the relationship between the sagittal plane COM and the calf muscles, while a different correlation was found between vCOMacc and calf muscle. Thus, it is possible that vCOMacc allows the observation of different aspects of balance control.

Note that the correlation between apCOMacc RMS and vCOMacc RMS was low. When apCOMacc is the result of multilinked joint control of the lower limbs [50], this suggests that vCOMacc is an index that can evaluate aspects of the physical control mechanism differently from apCOMacc. As the details of the mechanism giving rise to such characteristics have not yet been clarified, this is a desirable subject for future studies.

The present study had some limitations. This study relied upon an uncomplicated experiment, which specifically analyzed time variation in vCOMacc. One of the major objectives of this study was to investigate whether we could use the obtained data to identify age-related changes in postural control. However, it was not possible to clarify the cause of age-related differences in vCOMacc fluctuation. Additional measurements (e.g., heart rate and electroencephalogram recordings) could provide potential sources of time–frequency differences across age groups. In addition, the number of subjects who participated in this experiment is small, which may affect the accuracy of the estimation obtained in this experiment. Therefore, this experiment is positioned as a preliminary experiment for further research in the future.

In conclusion, a micro acceleration analysis in quiet standing using time–frequency characteristics revealed differing power spectral densities according to age. Time–frequency analysis showed multiple spectral activity peaks during body oscillations within each frequency range, indicating that the underlying signal contained within vCOMacc is highly dynamic. Therefore, the use of time–frequency methods, which provide information on not only the frequency content but also the timing of these dynamic features of spectral activity, is recommended for further investigation. Furthermore, vCOMacc was able to reflect the activity dynamics of lower limb muscle activities in the standing position, suggesting that neurological information in standing control may be collected using a simple method.

## Figures and Tables

**Table 1:**
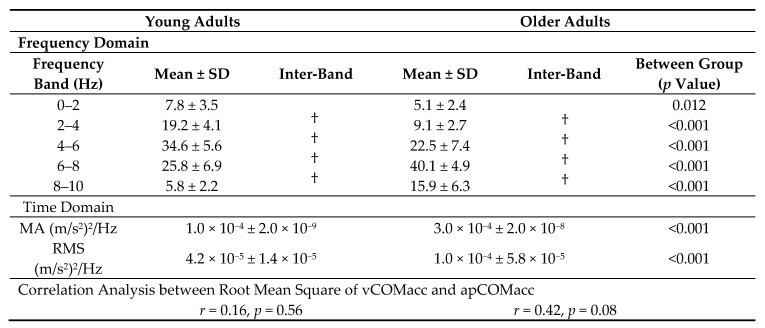
Summary of vertical center of mass acceleration (vCOMacc), integrated power spectral density (PSD), maximum amplitude (MA), root mean square (RMS), and correlation coefficient (*r*) for both age groups combined.

Values presented are means and standard deviations of the normalized power spectral density of each frequency band. Comparison of each frequency band, MA, and RMS differences between both groups. †, Statistical analysis within each frequency band. The last row of the table shows the correlation results between the root mean square of vCOMacc and apCOMacc. MA: maximum amplitude, RMS: root mean square.

**Table 2:**
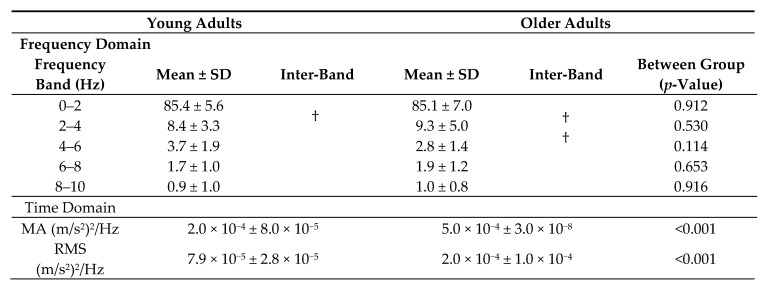
Summary of anterior-posterior center of mass acceleration (apCOMacc), integrated power spectral density, maximum amplitude, root mean square for both age groups combined.

Values presented are means and standard deviations of the normalized power spectral density of each frequency band. Comparison of frequency band, MA, and RMS differences between both groups. †, Statistical analysis within each frequency band.

**Table 3:** Correlation between the power spectral density of vCOMacc or apCOMacc in young adults and the root mean square of lower limb muscle activity.

Young Adults				
vCOMacc			apCOMacc	
Frequency Band (Hz)	MG RMS	So RMS	MG RMS	So RMS
0–2	−0.27	0.17	0.11	−0.41
2–4	−0.21	−0.18	−0.09	0.04
4–6	0.00	−0.62 **	−0.04	0.46 *
6–8	0.33	0.19	−0.06	0.71 **
8–10	−0.13	0.75 **	−0.19	0.69 **

MG: medial gastrocnemius, So: soleus; * *p* < 0.05, ** *p* < 0.01.

**Table 4:** Correlation between the power spectral density of vCOMacc or apCOMacc in older adults and the root mean square of lower limb muscle activity.

Older Adults				
vCOMacc			apCOMacc	
Frequency Band (Hz)	MG RMS	So RMS	MG RMS	So RMS
0–2	−0.61 **	−0.20	−0.26	−0.21
2–4	−0.43	0.13	0.07	0.06
4–6	−0.19	0.27	0.42	0.55 *
6–8	0.28	0.06	0.53 *	0.27
8–10	0.35	−0.27	0.34	0.18

MG: medial gastrocnemius, So: soleus; * *p* < 0.05, ** *p* < 0.01.
